# Yoga and lipid profile

**DOI:** 10.4103/0973-6131.78185

**Published:** 2011

**Authors:** Viroj Wiwanitkit

**Affiliations:** Wiwanitkit House, Bangkhae, Bangkok – 10160, Thailand

Sir,

I read with great interest the recent publication on yoga and lipid profile.[1] Acharya *et al*. concluded in their study that “*Pranayama* and *Yogasana* help in regulating sugar level also.”[1] This is an interesting finding. However, additional data are required to strengthen this finding. First, the sugar determination is highly affected by several pre-analytical and analytical interferences in laboratory medicine. Hence, good quality control is needed in laboratory analysis. Use of a more reliable laboratory test such as hemoglobin A1C may help in confirming the blood glucose-regulating effect of regular yoga practice. Second, a difficulty in performing such study is the control of external factors that can deviate the blood glucose results among subjects. These factors include difference in the lifestyle of each study subject.
